# Direct and tunable modulation of protein levels in rice and wheat with a synthetic small molecule

**DOI:** 10.1111/pbi.12787

**Published:** 2017-08-04

**Authors:** Jingbo Zhang, Kangquan Yin, Juan Sun, Jinlan Gao, Qiuli Du, Huali Li, Jin‐Long Qiu

**Affiliations:** ^1^ State Key Laboratory of Plant Genomics Institute of Microbiology Chinese Academy of Sciences Beijing China; ^2^ University of Chinese Academy of Sciences Beijing China; ^3^ Department of Life Science and Engineering Jining University Qufu China; ^4^ National Center for Soybean Improvement National Key Laboratory of Crop Genetics and Germplasm Enhancement Nanjing Agricultural University Nanjing China

**Keywords:** protein stability, small molecule, RDDK‐Shld1 system, wheat, rice

## Abstract

Direct control of protein level enables rapid and efficient analyses of gene functions in crops. Previously, we developed the RDDK‐Shield1 (Shld1) system in the model plant *Arabidopsis thaliana* for direct modulation of protein stabilization using a synthetic small molecule. However, it was unclear whether this system is applicable to economically important crops. In this study, we show that the RDDK‐Shld1 system enables rapid and tunable control of protein levels in rice and wheat. Accumulation of RDDK fusion proteins can be reversibly and spatio‐temporally controlled by the synthetic small‐molecule Shld1. Moreover, RDDK‐Bar and RDDK‐Pid3 fusions confer herbicide and rice blast resistance, respectively, in a Shld1‐dependent manner. Therefore, the RDDK‐Shld1 system provides a reversible and tunable technique for controlling protein functions and conditional expression of transgenes in crops.

## Introduction

A major scientific challenge in the postgenomic era is to characterize the function of all proteins, which often involves phenotypic analyses following perturbation or removal of a given protein. Perturbations of protein function can be achieved at the DNA, RNA and protein levels. In plants, gene knockout through T‐DNA or transposon insertion and newly developed genome editing technology have been widely used to study protein functions (Azpiroz‐Leehan and Feldmann, 1997; Martienssen, 1998; Voytas and Gao, 2014). RNA interference is also an important technique adapted to study protein function in plants through post‐transcriptional silencing (Small, 2007). However, to knockout a plant gene normally entails the regeneration and large‐scale screening of transgenic plants, which is time‐consuming and labour‐intensive. In addition, RNA interference often generates gene knockdowns in which proteins are only reduced to levels which are difficult to predict. RNAi of a gene can also be accompanied by nonspecific or off‐target effects (Stankunas and Crabtree, 2007). Furthermore, the gene knockouts and knockdowns created with these methods are irreversible, except that RNAi is coupled to an inducible promoter.

In contrast, perturbation of protein functions with chemicals is fast and typically reversible. Techniques based on small molecules have demonstrated their potential in probing gene functions and controlling transgene expression (Padidam, 2003). Several chemically inducible systems have been used to target and functionally analyse genes, including tetracycline repressor (TetR)‐based induction/inactivation (Gatz, 1997; Weinmann *et al*., 1994); glucocorticoid receptor‐based steroid induction (Lloyd *et al*., 1994; Picard, 1994); oestrogen receptor‐based steroid induction (Bruce *et al*., 2000; Zuo *et al*., 2000); AlcR‐based ethanol induction (Caddick *et al*., 1998); and ACEI‐based copper induction (Mett *et al*., 1993; Wright *et al*., 1988). However, these chemically inducible systems rely on regulating target gene mRNA levels. They therefore indirectly modulate protein function and may be compromised by protein stability and post‐translational modifications. Methods to directly perturb protein levels are thus much needed but limited in plants.

Certain small molecules can be used to directly, rapidly and reversibly regulate protein functions (Bain *et al*., 2003; Davies *et al*., 2000; Godl *et al*., 2003). For example, ATP analogs such as NM‐PP1 specifically inhibit directed mutant forms of protein kinases (Bishop *et al*., 2000). An alternative strategy is based on a mutant destabilizing domain (DD) of the FK506‐ and rapamycin‐binding protein (Clackson *et al*., 1998). Fusions of this unstable DD to proteins of interest (POIs) are rapidly degraded via the 26S proteasome. A synthetic cell‐permeable derivative of rapamycin, Shield1 (Shld1), binds the DD with high affinity to stabilize the fusion protein in a rapid, dose‐dependent and reversible manner (Banaszynski *et al*., 2006). This DD‐Shld1 system has been successfully used in various cell types and organisms including *Toxoplasma gondii, Plasmodium falciparum, Caenorhabditis elegans and Leishmania major* (Cho *et al*., 2013; Dvorin *et al*., 2010; Herm‐Götz *et al*., 2007; Madeira da Silva *et al*., 2009).

Recently, we adapted the DD‐Shld1 system in the model plant Arabidopsis (Su *et al*., 2013). However, our initial Arabidopsis DD‐POI or POI‐DD fusions were insufficiently unstable in the absence of ligand such that leaky protein accumulation was detected and the degree of leakiness was correlated with the expression level of the fusion gene. Therefore, we added extra instability determinants to the system by adding to the DD an N‐terminal arginine (R) residue, as per the protein stability N‐end rule (Bachmair *et al*., 1986) and a C‐terminal lysine (K) for proteosomal targeting of fusions to produce RDDK (Su *et al*., 2013). RDDK was also N‐terminally fused to ubiquitin which is rapidly excised post‐translationally in cells to expose the terminal arginine on the RDDK‐POI fusion. This modified RDDK‐Shld1 system displayed no background accumulation irrespective of transgene expression levels. Moreover, functional activities of RDDK fusions were induced in a Shld1‐dependent manner, confirming proof‐of‐concept of the RDDK‐POI system (Su *et al*., 2013). Whether the RDDK‐Shld1 system works in other plant species, especially monocot crops, remains to be tested.

Rice and wheat are two major crops worldwide. The rice genome has been sequenced, and a whole genome assembly for bread wheat has been produced. While rice is used as a model monocot due to its relatively small diploid genome (0.45 Gb), wheat is unusually recalcitrant to genetic analyses due to its allohexaploidy and large genome (17.1 Gb) (Dvořák, 2009). Currently, very few chemically inducible systems have been established in rice and wheat (Hirose *et al*., 2012; Ouwerkerk *et al*., 2001), and these systems target genes at the level of DNA and RNA. Therefore, the RDDK‐Shld1 system is a promising tool for probing gene functions in crops. In this study, we show that the RDDK‐Shld1 system is effective in both rice and wheat and that the functions of RDDK‐fused exogenous and endogenous proteins can be controlled in a Shld1‐dependent manner. Moreover, accumulation of RDDK fusions can be modulated spatially and temporally by Shld1. Our work provides a useful tool to investigate protein function directly and to control transgene expression in monocot crops.

## Results

### Tunable control of protein accumulation with the RDDK‐Shld1 system in rice

**Figure 1:**
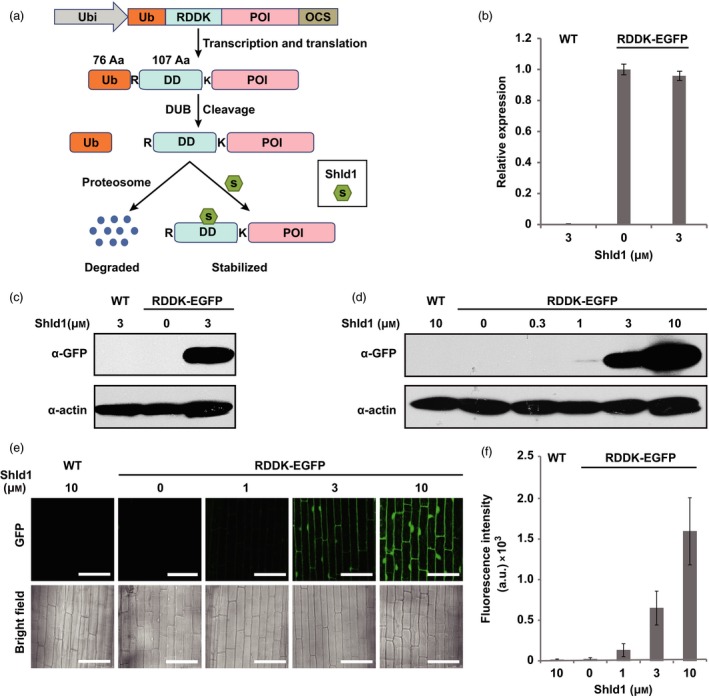
Shld1‐dependent accumulation of RDDK‐EGFP in rice. (a) Schematic of the RDDK–Shld1 system. Ub‐RDDK fusion gene is driven by maize *ubiquitin* promoter. During translation, ubiquitin fusion (Ub) is rapidly cleaved by endogenous deubiquitinating enzymes (DUBs) exposing the N‐terminal arginine (R). A lysine (K) is included as a potential recipient for ubiquitination of the fusion protein just after the destabilizing domain (DD). RDDK‐POI fusion proteins will thus be degraded by the 26S proteasome. Shld1 binds specifically to the DD domain such that the RDDK‐POI is stabilized. Ubi, maize *ubiquitin* promoter; POI, protein of interest; OCS, octopine synthase terminator; R, arginine; K, lysine. (b) Quantitative RT‐PCR analysis of *RDDK‐EGFP* fusion gene expression. Wild‐type (WT) and RDDK‐EGFP transgenic rice plants were treated with 3 μm Shld1 or mock treated for 8 h. Results were normalized to rice *UBIQUITIN 5* (Os*UBQ5*), and expression level of the transgene in mock‐treated RDDK‐EGFP transgenic rice plants was set at one unit. Error bars indicate SD. (c) Immunoblotting with anti‐GFP antibody detects Shld1‐induced accumulation of RDDK‐EGFP in the transgenic rice plants. Plants were treated with 3 μm Shld1 or mock solution for 8 h. ACTIN was used as a protein loading control. (d) Shld1 dose‐dependent accumulation of RDDK‐EGFP detected by immunoblotting with anti‐GFP antibody. RDDK‐EGFP plants were treated with varying concentrations of Shld1 for 8 h. (e) Confocal images of epidermal cells of leaf sheaths from wild‐type (WT) and RDDK–EGFP plants treated with varying concentrations of Shld1 for 8 h. Bars = 50 μm. (f) Fluorescence intensity quantification of the confocal microscopy images in (e). Error bars indicate SD. a.u., arbitrary unit.

To test whether RDDK‐Shld1 system was also functional in rice, we generated a vector based on pCambia2300 to express an *RDDK‐EGFP* fusion gene driven by the maize *ubiquitin* (*Ubi*) promoter (Christensen *et al*., 1992) (Figure 1a). Agrobacterium‐mediated transformation was used to generate RDDK‐EGFP transgenic rice plants. Forty independent transgenic lines were obtained and confirmed by PCR. In T1 generation, nine lines showed 3:1 segregation of the transgene. Among them, three lines were advanced to T2 generation. Finally, three homozygous T2 transgenic lines (e4, e15 and e35) were generated (Table S1). Rice plants without transgenes that segregated from RDDK‐EGFP transgenic lines were used as wild‐type (WT) controls. We found that *RDDK‐EGFP* transcript levels were similar in the three lines (Figure S1a) and accumulated similar levels of RDDK‐EGFP protein upon Shld1 treatment (Figure S1b). Line e15 was used for further studies. We then checked whether *RDDK‐EGFP* transcript in the transgenic plants was affected by application of Shld1. As expected, *RDDK‐EGFP* mRNA levels were unaffected by Shld1 treatment (Figure 1b). In contrast, GFP immunoblotting showed that RDDK‐EGFP fusion protein accumulated to high levels in response to 8 h of 3 μm Shld1 treatment in RDDK‐EGFP transgenics but not in the treated wild‐type or untreated RDDK‐EGFP transgenic controls (Figure 1c). These results indicate that Shld1‐induced RDDK fusion protein accumulation is highly specific.

Next, we investigated whether accumulation of RDDK‐EGFP fusion protein depends on Shld1 dosage in rice. RDDK‐EGFP transgenic rice seedlings were treated with various concentrations of Shld1 for 8 h. Total protein of treated tissues was then extracted and analysed by immunoblotting using an anti‐GFP antibody. As shown in Figure 1d, accumulation of RDDK‐EGFP was observed upon treatment with 1 μm Shld1, but increased to higher levels with more Shld1 applied and rose to a robust level upon 10 μm Shld1 treatment. That accumulation of RDDK‐EGFP fusion is controlled by Shld1 in dose‐dependent manner was further confirmed by quantifying the fluorescence intensity of confocal images (Figure 1e and f). This showed that Shld1 could stabilize RDDK fusions in rice and that the accumulation of the RDDK fusion was tunable by adjusting the amounts of applied Shld1.

### Temporal and spatial control of RDDK fusion accumulation with Shld1 in rice

**Figure 2:**
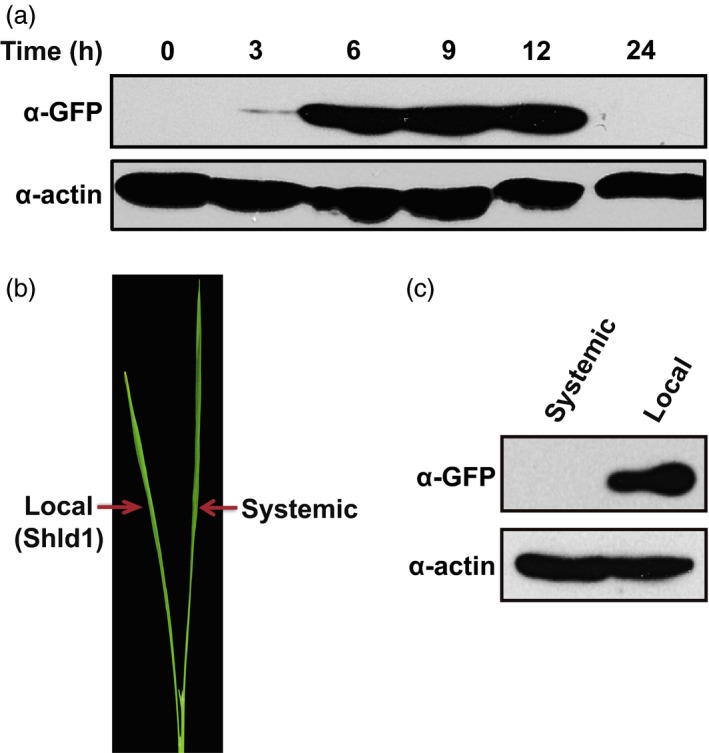
Temporal and spatial control of RDDK‐EGFP accumulation by Shld1 in rice. (a) Shld1‐induced RDDK‐EGFP accumulation is reversible in rice. 14‐day‐old rice plants were first treated with 3 μm Shld1 and washed 3 h later with water to remove Shld1. Leaves were collected at the indicated times of Shld1 application with ‘0’ as leaves collected just before Shld1 application. Levels of RDDK‐EGFP were detected by immunoblotting with anti‐GFP antibody. (b) A representative picture showing 3 μm Shld1‐treated local leaf and systemic leaf without Shld1 treatment on a rice seedling. (c) Immunoblotting with anti‐GFP antibody of total protein extracts from local and systemic leaves as in (b).

To analyse the kinetics of RDDK fusion accumulation, RDDK‐EGFP transgenic rice plants were first treated with 3 μm Shld1 for 3 h, and then Shld1 on the surface was removed by washing the plants with water. Accumulation of RDDK‐EGFP over time was analysed by immunoblotting using an anti‐GFP antibody. RDDK‐EGFP protein was barely detected 3 h after Shld1 treatment. The accumulation of RDDK‐EGFP protein increased over time with maximum accumulation within 9 h. After that protein levels started to drop, and by 24 h the fusion protein was undetectable, suggesting that modulation of RDDK fusions by Shld1 is reversible in rice (Figure 2a). Shld1 effects are apparently much slower in rice than in Arabidopsis (Su *et al*., 2013), most probably due to differences in their epidermal permeability to Shld1. Specific control of RDDK fusion protein accumulation by Shld1 was further investigated. We treated one leaf of the RDDK‐EGFP transgenic plants with 3 μm Shld1 for 8 h and then assayed accumulation of RDDK‐EGFP in the treated (local) and untreated (systemic) leaves (Figure 2b). This revealed that RDDK‐EGFP protein only accumulated in leaves treated with Shld1, as it was undetectable in systemic leaves (Figure 2c). These data confirm that the RDDK‐Shld1 system provides spatial and temporal control of RDDK fusion proteins in rice.

### Shld1‐induced herbicide resistance in RDDK‐Bar transgenic rice

**Figure 3:**
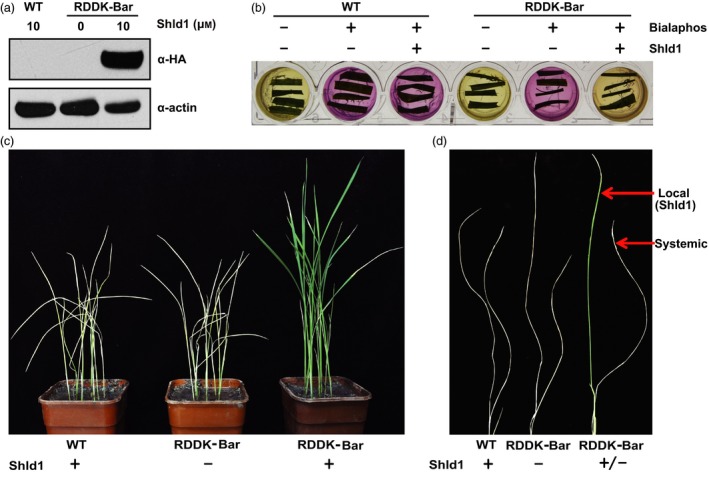
Shld1‐induced herbicide resistance in RDDK‐Bar transgenic rice plants. Shld1‐induced accumulation of RDDK‐Bar fusion protein in rice. Immunoblotting with anti‐HA antibody of total extracts from WT and RDDK‐Bar transgenic rice plants treated with or without 10 μm Shld1 for 8 h. ACTIN was used as a protein loading control. (b) Shld1‐induced bialaphos resistance of RDDK‐Bar plants as shown by the CR assay. Leaf pieces of WT and RDDK‐Bar plants were excised and cultured separately in a 24‐well plate with CR medium supplemented with (‘+’) or without (‘−’) 8 mg/L bialaphos and/or 10 μm Shld1. The plate was incubated in a growth chamber at 24 °C with a light/dark cycle of 16 h/8 h. Photographs were taken 3 days after treatment. (c) Basta resistance of rice plants induced by Shld1. 14‐day‐old WT and RDDK‐Bar plants were first treated with or without 10 μm Shld1 for 3 h before spraying once with Basta. Photographs were taken 10 days after treatment. (d) Shld1 conferred spatial control of herbicide resistance in RDDK‐Bar transgenic rice plants. Local leaf of a 14‐day‐old RDDK‐Bar plant was treated with 10 μm Shld1; 3 h later, both local and systemic leaves were sprayed with Basta. Shld1‐treated WT plants and mock‐treated RDDK‐Bar plants were sprayed with Basta as controls. Photographs were taken 10 days after treatment.

Herbicide resistance genes are important for generating herbicide‐tolerant crops which offer a vital tool in fighting weeds and preserving topsoil. Most commercial herbicide‐resistant crops contain transgenes originated from other organisms. One strategy to mitigate concerns about these genetically modified crops is to make herbicide resistance gene inducible. We reasoned that the RDDK‐Shld1 system is an ideal tool for this strategy. To test this, we constructed an *RDDK‐Bar* fusion tagged with the HA epitope, driven by the maize *ubiquitin* (*Ubi*) promoter, and transformed it via *Agrobacterium tumefaciens* into rice callus. Twenty‐nine independent transgenic lines were obtained and confirmed by PCR. In T1 generation, nine lines demonstrated 3:1 segregation of the transgene. Among them, three lines were advanced to T2 generation. Finally, three homozygous T2 transgenic lines (b11, b13 and b19) were obtained (Table S1). Rice plants without transgenes that segregated from RDDK‐Bar transgenic lines were used as wild‐type (WT) controls. Levels of RDDK‐Bar accumulated after 10 μm Shld1 treatment appear to correlate with *RDDK‐Bar* transcript levels in the three lines, of which line b19 exhibited highest RDDK‐Bar accumulation upon Shld1 application (Figure S2a,b). We used line b19 for further studies. We treated the RDDK‐Bar transgenic and WT plants with 10 μm Shld1 for 8 h and used mock‐treated RDDK‐Bar transgenic rice plants as a negative control. Immunoblotting analysis using anti‐HA antibody revealed that Shld1 stabilized the RDDK‐Bar protein in rice plants (Figure 3a).

The *Bar* gene from *Streptomyces hygroscopicus* encodes a phosphinothrincin acetyltransferase (PAT) which converts phosphinothrincin (PPT) into a nontoxic acetylated form and confers plants resistance to glufosinate‐based herbicides (Botterman *et al*., 1991). PPT inhibits glutamine synthetase (GS) and disturbs ammonia (NH_3_) assimilation in plant cells. The chlorophenol red (CR) assay takes advantage of the pH change caused by ammonia accumulation upon GS inhibition (Gao *et al*., 2006; Kramer *et al*., 1993). We tested whether Shld1‐induced RDDK‐Bar was functional in rice using a leaf CR assay. Leaf pieces of RDDK‐Bar transgenic and WT rice plants were incubated at room temperature in the CR medium supplied with various combinations of bialaphos (8 μg/mL) and 10 μm Shld1. Bialaphos is a naturally occurring herbicide made up of a PPT moiety and two alanine residues, which is metabolized into PPT in plants by the action of a peptidase (Duke, 1996). Bialaphos treatment alone changes the CR medium with leaf pieces from RDDK‐Bar transgenic rice plants to purple. However, when leaf pieces from RDDK‐Bar transgenic rice plants were treated with bialaphos and Shld1 together, the CR medium changes to yellow. In contrast, WT leaves treated with both bialaphos and Shld1 turn the CR medium to purple (Figure 3b). These data indicate that the RDDK‐Bar fusion protein is functional in rice and its activity can be modulated in a Shld1‐dependent manner.

Next, we tested whether Shld1 was able to induce Basta resistance in RDDK‐Bar transgenic rice plants. The RDDK‐Bar transgenic and WT rice seedlings were pretreated with 10 μm Shld1 and 3 h later were sprayed with 1:180 dilution of the commercial herbicide Basta (containing 180 g/L glufosinate ammonium, Bayer CropScience). As a control, mock‐treated RDDK‐Bar transgenic rice plants were also sprayed with Basta. While the Shld1‐treated WT plants and mock‐treated RDDK‐Bar transgenic rice plants died 10 days after Basta application, Shld1‐treated RDDK‐Bar transgenic rice plants showed almost no death symptoms (Figure 3c). We performed the same experiment on the other two lines and obtained similar results (Figure S2c). These results confirm that RDDK‐Bar is stabilized by Shld1 and that the resulting levels of PAT activity are sufficient to confer herbicide resistance. We also tested the spatial control of RDDK‐Bar function by Shld1. Single leaves of RDDK‐Bar transgenic plants were treated with 10 μm Shld1. As controls, WT plants were also treated with 10 μm Shld1 and another batch of RDDK‐Bar transgenic plants were treated with a mock solution. After 3 h, leaves of these plants were sprayed with Basta. As shown in Figure 3d, only Shld1‐treated leaves of RDDK‐Bar transgenic plants survived, remaining green 10 days after Basta application. This confirms that RDDK‐Bar‐conferred herbicide resistance can be spatially controlled by Shld1.

### Shld1‐dependent blast resistance in RDDK‐Pid3 transgenic rice

**Figure 4:**
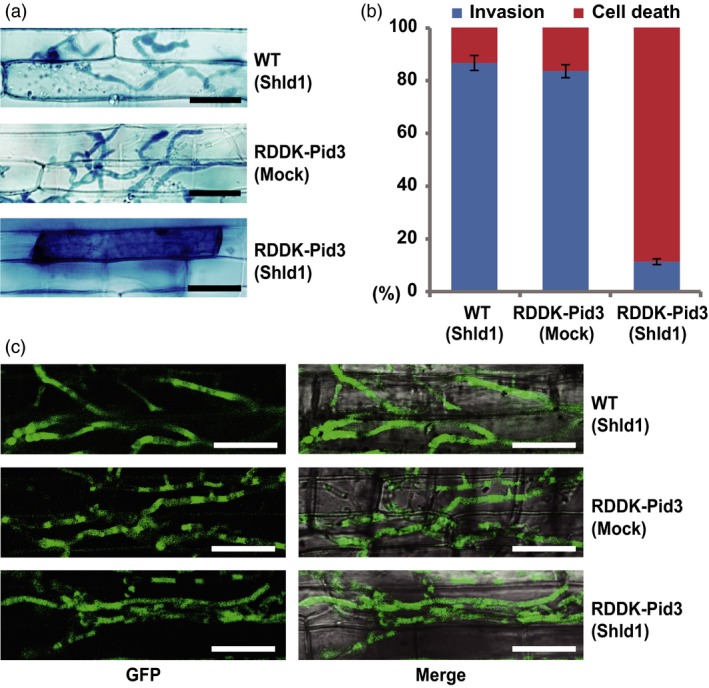
Shld1‐induced race‐specific resistance of rice to the blast fungus *Magnaporthe oryzae*. Shld1‐dependent resistance of RDDK‐Pid3 plants to an avirulent strain of *M. oryzae*. Leaf sheaths were detached from 4‐week‐old rice plants and inoculated with a suspension of 2 × 10^5^ mL^−1^ fungal spores of *M. oryzae* strain Zhong‐10‐8‐14 with 10 μm Shld1 (indicated as ‘Shld1’) or without Shld1 (indicated as ‘Mock’). Shown are bright‐field images of sheath cells 48 h postinfection stained with Trypan blue to highlight the fungus and host cell death. Bars = 25 μm. (b) Quantitative analysis of compatible and incompatible interactions of the rice sheath cells in the same experiment as in (a). (c) Shld1‐treated RDDK‐Pid3 plants remain susceptibility to virulent *M. oryzae* strain. Shown are confocal images of sheath cells 48 h postinfection with a GFP‐tagged *M. oryzae* strain ZB15. Representative images are shown. Bars = 25 μm.

To further test the RDDK‐Shld1 system with an endogenous rice protein, we generated RDDK‐Pid3 transgenic rice in ‘Nipponbare’ (*japonica*) background. Twenty‐seven independent transgenic lines were obtained and confirmed by PCR. In T1 generation, eight lines showed 3:1 segregation of the transgene. Among them, three lines were advanced to T2 generation. Finally, three homozygous T2 transgenic lines (p4, p5, and p6) were generated (Table S1). We found that *RDDK‐Pid3* transcript levels were similar in them (Figure S3a) and used line p5 for further studies. *Pid3* encodes a nucleotide binding, leucine‐rich repeat (NB‐LRR) protein and confers race‐specific resistance to *Magneportha oryzae*. Notably, the *Pid3* alleles in most *japonica* varieties (including ‘Nipponbare’) are pseudogenes due to a non‐sense mutation in the LRR region (Shang *et al*., 2009). We first analysed blast resistance using a leaf sheath inoculation assay with spores of *M. oryzae* strain Zhong‐10‐8‐14, which is virulent on Nipponbare (Lv *et al*., 2013; Shang *et al*., 2009). Leaf sheaths of RDDK‐Pid3 transgenic and WT plants were inoculated with spores of *M. oryzae,* and 10 μm Shld1 was omitted or added in the spore suspensions. Trypan blue staining was used to visualize fungal structures and rice cell death 48 h postinfection. We found that 86.6% of the appressoria formed on WT plants invaded host cells and formed invasive hyphae (IH) (Figure 4a and b), indicating Nipponbare is susceptible to this strain. In contrast, 88.7% of infected cells of Shld1‐treated RDDK‐Pid3 transgenic plants were dead, indicating their induction of the immune hypersensitive response (HR) to the *M. oryzae* strain Zhong‐10‐8‐14 (Figure 4a and b). However, mock‐treated RDDK‐Pid3 transgenic plants were as susceptible as WT plants to the *M. oryzae* strain (Figure 4a and b). We performed the same experiment on the other two lines and obtained similar results (Figure S3b). To further confirm that the Shld1‐induced blast resistance of RDDK‐Pid3 transgenic plants is race specific, we tested the resistance to *M. oryzae* strain ZB15. This strain is virulent on rice harbouring functional *Pid3* (Lv *et al*., 2013; Shang *et al*., 2009). A GFP‐tagged ZB15 strain was used for the leaf sheath assay. As expected, fungal appressoria formed IH in most of the infected cells of Shld1‐treated RDDK‐Pid3 transgenic plants, similar to that of WT and mock‐treated RDDK‐Pid3 transgenic plants (Figure 4c). These data indicate that Shld1 specifically induces RDDK‐Pid3 accumulation in the leaf sheath and thus confers race‐specific resistance to the rice blast fungus.

### Modulation of protein accumulation with the RDDK‐Shld1 system in wheat

**Figure 5:**
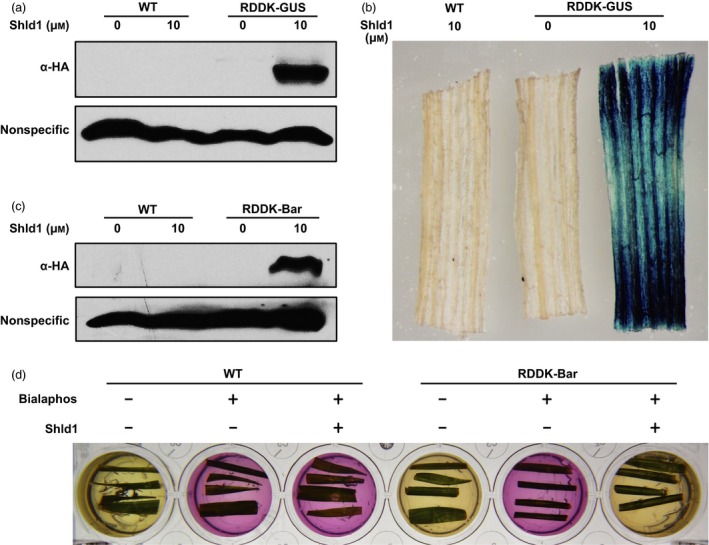
Modulation of protein levels and function with the RDDK‐Shld1 system in wheat. Shld1‐induced accumulation of RDDK‐GUS fusion protein in wheat. Immunoblotting with anti‐HA antibody of total extracts from RDDK‐GUS transgenic and WT wheat plants. Plants were treated with 10 μm Shld1 or mock solution for 8 h. (b) GUS staining of leaf pieces of RDDK‐GUS and WT plants treated with 10 μm Shld1 or mock solution for 8 h. A representative image is shown. (c) Shld1‐induced accumulation of RDDK‐Bar fusion protein in wheat. Immunoblotting with anti‐HA antibody of total extracts from RDDK‐Bar and WT plants treated with 10 μm Shld1 or mock solution for 8 h. (d) Shld1‐induced bialaphos resistance of RDDK‐Bar transgenic wheat as shown by CR assay. Leaf pieces of WT and RDDK‐Bar transgenic wheat plants were excised and cultured separately in a 24‐well plate with CR medium supplemented with (‘+’) or without (‘−’) 8 mg/L bialaphos and/or 10 μm Shld1. The plate was incubated in a growth chamber at 24 °C with a light/dark cycle of 16 h/8 h. Photographs were taken 3 days after treatment.

Following our demonstration that the RDDK‐Shld1 system works in rice, we tested whether it also functions in another important monocot crop. To this end, we generated transgenic bread wheat using a transformation vector based on pJIT163Ubi (Wang *et al*., 2014) in which an HA‐tagged *RDDK‐GUS* fusion gene was driven by the maize *Ubi* promoter. T2 plants homozygous for the transgene were used for further experiments (Table S1). Wild‐type (WT) wheat plants segregated from the transgenic lines were used as control. The RDDK‐GUS transgenic and WT wheat plants were treated with mock solution or with 10 μm Shld1 for 8 h. Immunoblotting using anti‐HA antibody revealed that RDDK‐GUS fusion protein accumulated in RDDK‐GUS transgenic wheat plants in the presence of Shld1 (Figure 5a). Next, we tested whether the stabilized RDDK‐GUS fusion protein is functional. GUS staining assay showed that Shld1‐treated leaves of RDDK‐GUS transgenic plants displayed blue coloration, confirming that the RDDK‐GUS protein was functional and dependent on Shld1 (Figure 5b).

### Shld1‐induced herbicide resistance in RDDK‐Bar transgenic wheat

To further test whether the RDDK‐Shld1 system is able to modulate wheat phenotypes in a Shld1‐dependent manner, we generated RDDK‐Bar (HA‐tagged *RDDK‐Bar* fusion gene) transgenic wheat plants. Thirty‐nine independent transgenic lines were obtained and confirmed by PCR. In T1 generation, eight lines exhibited 3:1 segregation of the transgene. Among them, three lines were advanced to T2 generation. Finally, three homozygous T2 transgenic lines (w2, w3 and w5) were obtained (Table S1). Wheat plants without transgenes that segregated from RDDK‐Bar transgenic lines were used as wild‐type (WT) controls. As observed in RDDK‐Bar transgenic rice plants, we found that RDDK‐Bar protein levels accumulated after 10 μm Shld1 treatment were also positively correlated with *RDDK‐Bar* transcript levels in the three lines, of which line w5 showed highest RDDK‐Bar accumulation upon Shld1 application (Figure S4a,b). Line w5 was used for further experiments. We then treated this RDDK‐Bar transgenic line and WT wheat plants with mock or with 10 μm Shld1 for 8 h. Immunoblotting using anti‐HA antibody showed that Shld1 induced RDDK‐Bar protein accumulation. In contrast, WT plants with Shld1 treatment, as well as RDDK‐Bar transgenic plants with mock treatment, did not show RDDK‐Bar protein accumulation (Figure 5c). This confirms that Shld1 stabilizes RDDK fusion protein specifically in wheat. The leaf CR assay was used to test modulation of herbicide resistance in the transgenic wheat plants by Shld1. Leaf pieces of RDDK‐Bar transgenic wheat plants and WT plants were incubated in CR medium with or without bialaphos and 10 μm Shld1 to check their diagnostic colour change. Leaf pieces from the RDDK‐Bar transgenic wheat plants treated with both bialaphos and Shld1 turned the CR medium yellow, indicating that stabilized by Shld1, RDDK‐Bar was able to detoxify bialaphos. Meanwhile, leaf pieces from RDDK‐Bar transgenic wheat plants treated only with bialaphos turned the CR medium to purple (Figure 5d). We performed the same experiment with the other two lines and obtained similar results (Figure S4c). We conclude that the accumulation and function of RDDK fusions can be modulated in a Shld1‐dependent manner in hexaploid bread wheat.

## Discussion

The genomes of rice and wheat have been sequenced, and the elucidation of all of their gene functions is now a pressing task. A system for conditional control of protein stability would be a useful tool to fulfil this task. In this work, we report on application of the RDDK‐Shld1 system for spatio‐temporal control of protein accumulation in monocot crops. We demonstrate that biological functions of the RDDK fusions can be modulated in a Shld1‐dependent manner in rice and wheat. Therefore, the RDDK‐Shld1 system provides a powerful tool for basic and applied research in monocot crops.

A robust, inducible gene expression system has several characteristics including rapid induction, reversibility, high stringency and dose dependence. The DD‐Shld1 system was established in mammalian cells for tunable control of protein levels with a small synthetic molecule ligand Shld1 (Banaszynski *et al*., 2006). A limitation of the DD‐Shld1 system was its leakiness, as shown in *P. falciparum* and *Arabidopsis* (Armstrong and Goldberg, 2007; De Azevedo *et al*., 2012; Su *et al*., 2013). To circumvent this problem, we exploited the general N‐end rule to develop the RDDK‐Shld1 system. As a proof‐of‐principle, we demonstrated in *Arabidopsis* that the RDDK‐Shld1 system displays almost no leakiness (Su *et al*., 2013). Here, we applied the RDDK‐Shld1 system in rice and wheat and did not detect any leakiness of transgenic proteins by immunoblotting. Thus, the stringency of the RDDK‐Shld1 system is maintained in monocot plants. We also demonstrated reversible control of protein accumulation with this system in rice as accumulation of RDDK‐EGFP diminished 24 h after Shld1 treatment in rice seedlings (Figure 2a). We note that in NIH3T3 fibroblasts, DD‐YFP fluorescence decreased rapidly from 1 h after withdraw of Shld1 (Banaszynski *et al*., 2006). This apparent temporal difference in reversibility may be due to the retention of Shld1 by the cell walls and epidermal cuticles of rice cells versus the rapidity of washing Shld1 from cultured mammalian cells.

RDDK‐EGFP accumulated in rice within 3 h of treatment with 3 μm Shld1 (Figure 2), whereas in Arabidopsis, RDDK‐EGFP began to accumulate as early as 0.5 h (Su *et al*., 2013). This difference is likely due to a lower permeability of rice versus Arabidopsis leaves. Nevertheless, this accumulation is more rapid than those induced by other inducible chemical systems such as the oestrogen‐inducible system (Zuo *et al*., 2000) in which transgenic GFP signal in rice scan be detected 48 h after estradiol treatment (Okuzaki *et al*., 2011). We also showed Shld1 dose‐dependent accumulation of RDDK‐POI fusion proteins from constitutively expressed transgenes (Figure 1). In addition, plants expressing the exogenous *Bar* gene and the endogenous resistance gene *Pid3* fused with *RDDK* exhibited herbicide resistance and race‐specific disease resistance only upon Shld1 application. Taken together, the RDDK‐Shld1 system provides a useful tool for directly modulating protein function in plants. Although we have modified the original DD domain, the RDDK‐Shld1 system maintains its characteristics of speed, dose dependence and reversibility. Considering its high stringency, the RDDK‐Shld1 system is also a promising tool in other eukaryotic species.

We also note here that localized application of Shld1 to RDDK‐Bar transgenic rice induced herbicide resistance in local but not systemic tissues. Such stringent spatio‐temporal control of protein function has a number of applications, particularly for proteins such as plant resistance (R) proteins whose constitutive expression is deleterious (Zhang *et al*., 2003).

Genetically modified crops can significantly increase agronomic yield and quality and reduce pesticide use (Huang *et al*., 2005). Constitutive promoters are often used to drive the expression of transgenes throughout tissues and developmental stages. However, constitutive expression of gene products, particularly those conferring increased disease resistance and stress tolerance, may have deleterious effects on growth and yield (Zhu *et al*., 2010). If the transgene is controlled with the RDDK‐Shld1 system, it might 1) enable growers to cheaply and rapidly induce responses to disease or stress when needed and 2) be rapidly reversible to return plants to normalized physiology and yields. As a proof‐of‐concept, we used RDDK‐Shld1 system to control RDDK‐Bar in rice and wheat. Both in rice and wheat, RDDK‐Bar protein accumulation and herbicide resistance can be specifically controlled by Shld1. These results indicate that the RDDK‐Shld1 system is an ideal tool for conditional control of transgene products in crops.

Despite its apparent advantages and potential uses discussed above, at least two limitations remain on the practical use of the RDDK‐Shld1 system in plants. First, the RDDK domain, which incorporates the N‐end rule, can only be added to the N terminus of proteins, a constraint for proteins of interest whose functions and/or targeting require unobstructed N‐termini. This limitation could be overcome by an alternative C‐terminal degron such as that of mouse ornithine decarboxylase (Li and Coffino, 1993; Takeuchi *et al*., 2008). Second, we used two different surfactants for delivering Shld1 into dicot and monocot plant species. A surfactant can be a double‐edged sword in that higher surfactant concentrations (*e.g*. Silwet L‐77) which reduce surface tension to increase Shld1 entry into cells also exhibit higher toxicity to plant cells (Whalen *et al*., 1991). Moreover, as different plants have different cell surface structures, optimal surfactants, as well as the permeability of Shld1 analogs, may require tailoring for different crops.

## Experimental procedures

### Plant growth conditions

Seedlings of rice (*Oryza sativa* L. ssp. *japonica*. cv. Nipponbare and cv. Zhonghua 11) were grown on soil in a controlled growth chamber at 28 °C with a 13‐h light/11‐h dark cycle and at 85% relative humidity. Seedlings of wheat (*Triticum aestivum*) were grown on soil in a controlled growth chamber at 22 °C with a 16‐h light/8‐h dark cycle.

### Plasmid construction

*RDDK‐EGFP* coding region was PCR amplified from the vector pRDDK‐EGFP (Su *et al*., 2013) and cloned into pCAMBIA2300‐Ubi in which the maize *Ubiquitin* promoter was inserted to drive transgene expression. The resulting construct was named pUbi:RDDK‐EGFP. The coding sequence of *RDDK‐HA* was PCR amplified from pRDDK‐HA (Su *et al*., 2013) and inserted into pCAMBIA2300‐Ubi to generate pUbi:RDDK‐HA. *Bar*,* GUS* and *Pid3* (Shang *et al*., 2009) coding regions were amplified by PCR with linker and inserted in frame with *RDDK‐HA* in pUbi:RDDK‐HA to generate the *RDDKHA‐Ba*r, *RDDKHA‐GUS* and *RDDKHA‐Pid3*. For wheat transformation, the *RDDKHA‐Bar* and *RDDKHA‐GUS* fusion genes were subcloned into pJIT163Ubi (Wang *et al*., 2014). Primers used for plasmid construction are listed in Table S1.

### Plant transformation and selection of homozygous transgenic plants

*Agrobacterium*‐mediated rice transformation was performed as previously described (Hiei *et al*., 1994). Binary vectors were first transformed into *Agrobacterium tumefaciens* AGL1 strain. *Agrobacterium* containing *RDDK‐EGFP* or *RDDK‐Bar* were then transformed into embryonic callus of *japonica* rice from rice cultivar Zhonghua No. 11 (ZH11). *RDDK‐Pid3* was transformed into embryonic callus of *japonica* rice variety Nipponbare. Transgenic plants were then selected and regenerated on G418‐containing medium.

Biolistic transformation of wheat immature embryos was performed as previously described (Wang *et al*., 2014; Zhang *et al*., 2015). Immature embryos of hexaploid bread wheat (*Triticum aestivum*) cultivar Kenong199 were used for transformation. Regenerated T0 transgenic wheat plants were transferred to soil and grown in growth chambers.

PCR amplification of the RDDK fusion gene was performed to genotype segregants of the transgenic rice and wheat plants. Transgenic lines with a ratio of 3:1 (RDDK fusion gene^+^: RDDK fusion gene^‐^) in the T1 generation were chosen for further analyses. T2 plants homozygous for the transgenes were used in this study. Primers used are listed in Table S2.

### Shld1 treatment of seedlings

Fourteen‐day‐old rice or wheat seedlings were sprayed with indicated concentrations of Shld1 (Clontech) in 0.05% BREAK‐THRU^®^ S 233 (Evonik) and then incubated in a chamber with 80%–90% humidity.

### RNA isolation, semi‐quantitative RT‐PCR and quantitative RT‐PCR

Total RNA was extracted with TRNzol reagent (Tiangen, Beijing) and then treated with RNase‐free DNase and reverse transcribed to first‐strand cDNA with PrimeScript RT reagent Kit (TaKaRa). For semi‐quantitative RT‐PCR, 25 μL reaction mixtures contained 0.5U of *Taq* DNA polymerase (Tiangen, Beijing), 200 μm of each dNTP, 0.2 μm of each primer and 80 ng cDNA. PCR parameters were as follows: 5 min at 95 °C, 28 cycles of 95 °C for 30 s, 60 °C for 30 s and 72 °C for 30 s. For quantitative RT‐PCR, SYBR Premix Ex Taq II (TaKaRa) was used to quantify the expression of each target gene using a Bio‐Rad CFX96 PCR System (Bio‐Rad Laboratories). Target gene expression levels were calculated by the 2^−ΔΔCt^ method using CFX Manager Software (Bio‐Rad). Primers used are listed in Table S2.

### Protein extraction and immunoblotting

Rice or wheat tissues were ground in liquid nitrogen, and total protein was extracted as previously described (Su *et al*., 2013). Immunoblotting was performed following standard procedures. Antibodies used were as follows: anti‐GFP (Roche, Cat#11814460001, 1:2500 dilution), anti‐HA (Roche, Cat#11583816001, 1:2500 dilution), anti‐Actin (Abmart, Cat# M20009L, 1:2000 dilution) and peroxidase‐conjugated goat anti‐mouse IgG secondary antibody (Sigma, Cat# A4416, 1:10 000 dilution).

### Confocal microscopy

A Leica TCS SP8 II microscopy was used to observe the GFP signal with excitation at 488 nm and emission at 515/530 nm. Relative fluorescence intensity was calculated with ImageJ software (http://rsb.info.nih.gov/ij/).

### Chlorophenol red (CR) assay

CR assays were performed as previously described (Gao *et al*., 2006; Kramer *et al*., 1993) with CR medium containing 1/2 strength MS salts, 8 g/L agar and 25 mg/L chlorophenol red, pH 6.0. Chlorophenol red, 10 μm Shld1 and 8 mg/L bialaphos (Wako Pure Chemical Industries) were added to the sterile culture medium before casting. 1‐cm‐long leaf tips were cut and put in CR medium in 24 multiwell plates and incubated in a chamber at 24 °C with 16‐h/8‐h light/dark photoperiod. Photographs were taken 3 days after incubation.

### Herbicide treatment of seedlings

1:180 dilution of the commercial herbicide Basta (containing 180 g/L glufosinate ammonium, Bayer CropScience) was used to spray rice seedlings. Plants were then grown in growth chamber with 80%–90% humidity. Photographs were taken 10 days after the treatment.

### *Magnaporthe oryzae* infection assay

Leaf sheath assay were performed as reported previously (Koga and Horino, 1984; Koga *et al*., 2004). Briefly, leaf sheaths of 4‐ to 5‐week‐old rice were inoculated with 2 × 10^5^ mL^−1^ *M. oryzae* strain Zhong‐10‐8‐14 or GFP‐tagged ZB15 (Shang *et al*., 2009) spores in 0.1% Tween 20 supplemented with or without 10 μm Shld1. To visualize fungal structure of *M. oryzae* strain Zhong‐10‐8‐14 and host cell death, sheath cells were stained with Trypan blue as previously described (Saitoh *et al*., 2012), and photographs were taken with an Olympus BX51 light microscope. Fungal structures of *M. oryzae* strain ZB15 were observed with a Leica TCS SP8 II confocal microscope.

### GUS staining

GUS staining was performed as previously described (Jefferson *et al*., 1987). Briefly, wheat leaves treated with mock solution or 10 μm Shld1 were incubated for 12 h at 37 °C in a phosphate‐buffered solution containing 5‐bromo‐4‐chloro‐3‐indolyl glucuronide (X‐Gluc; 1 mg/mL) and then were cleared with serial dilutions of ethanol.

## Funding

This work was supported by grants to J.‐L.Q. from the National Transgenic Science and Technology Program of China (2014ZX0801002B), National Key Research and Development Program of China (2016YFD0100602) and The Danish Council for Independent Research | Technology and Production Sciences (4184‐00019), and to Q.D. from the Natural Science Foundation of Shandong Province (ZR2012CM011).

## Supporting information


**Figure S1** Chracterization of RDDK‐EGFP transgenic rice plants.
**Figure S2** Chracterization of RDDK‐Bar transgenic rice plants.
**Figure S3** Chracterization of RDDK‐Pid3 transgenic rice plants.
**Figure S4** Chracterization of RDDK‐Bar transgenic wheat plants.
**Table S1** Transgenic lines generated in this study.
**Table S2** Sequences of the primers used in this work.
