# Supplementary material for: Direct and tunable modulation of protein levels in rice and wheat with a synthetic small molecule
Source: Plant Biotechnol J. 2017 Aug 4;16(2):472–81. doi: 10.1111/pbi.12787 (PMC5787845; doi:10.1111/pbi.12787)
Supplement: Supplementary file 1 — Figure S1 Chracterization of RDDK‐EGFP transgenic rice plants. Figure S2 Chracterization of RDDK‐Bar transgenic rice plants. Figure S3 Chracterization of RDDK‐Pid3 transgenic rice plants. Figure S4 Chracterization of RDDK‐Bar transgenic wheat plants. Table S1 Transgenic lines generated in this study. Table S2 Sequences of the primers used in this work. [file PBI-16-472-s001.docx]

**Supporting information**

**Figure S1.** Chracterization of RDDK-EGFP transgenic rice plants.

**Figure S2.** Chracterization of RDDK-Bar transgenic rice plants.

**Figure S3.** Chracterization of RDDK-Pid3 transgenic rice plants.

**Figure S4.** Chracterization of RDDK-Bar transgenic wheat plants.

**Table S1.** Transgenic lines generated in this study.

**Table S2.** Sequences of the primers used in this work.





**Figure S1.** Chracterization of RDDK-EGFP transgenic rice plants.

(a) Expression of *RDDK-EGFP* determined by semi-quantitative RT-PCR in the wild-type (WT), and three independent transgenic lines that homozygous for the *RDDK-EGFP* transgene (e4, e15 and e35). Levels of *ACTIN1* transcripts were used as loading controls.

(b) Shld1-induced accumulation of RDDK-EGFP fusion protein in the three independent transgenic lines. Immunoblotting with anti-GFP antibody of total extracts from WT and RDDK-EGFP transgenic rice plants treated with or without 10 μM Shld1 for 8 hours. ACTIN was used as a protein loading control.





**Figure S2.** Chracterization of RDDK-Bar transgenic rice plants.

(a) Expression of *RDDK-Bar* determined by semi-quantitative RT-PCR in the wild-type (WT), and three independent transgenic lines (b11, b13 and b19) that homozygous for the *RDDK-Bar* transgene. Levels of *ACTIN1* transcripts were used as loading controls.

(b) Shld1-induced accumulation of RDDK-Bar fusion protein in the three independent transgenic lines. Immunoblotting with anti-HA antibody of total extracts from WT and RDDK-Bar transgenic rice plants treated with or without 10 μM Shld1 for 8 hours. ACTIN was used as a protein loading control.

(c) Basta resistance of rice plants induced by Shld1. 14-day-old WT and RDDK-Bar plants of line b11 and b13 were first treated with or without 10 μM Shld1 for 3 hours before spraying once with Basta. Photographs were taken ten days after treatment.





**Figure S3.** Chracterization of RDDK-Pid3 transgenic rice plants.

(a) Expression of *RDDK-Pid3* determined by semi-quantitative RT-PCR in the wild-type (WT), and three independent transgenic lines (p4, p5 and p6) that homozygous for the *RDDK-Pid3* transgene. Levels of *ACTIN1* transcripts were used as loading controls.

(b) Shld1-dependent resistance of RDDK-Pid3 transgenic rice plants of line p4 and p6 to an avirulent strain of *Magnaporthe oryzae*. Leaf sheath were first detached from four-week-old rice plants, and inoculated with a suspension of 2×10^5^ ml^-1^ fungal spores of *M. oryzae* strain Zhong-10-8-14 with 10 μM Shld1 (indicated as “Shld1”) or without Shld1 (indicated as “Mock”). Shown are bright-field images of sheath cells 48 hours post infection stained with Trypan blue to highlight the fungus and host cell death. Bars = 25 μm.





**Figure S4.** Chracterization of RDDK-Bar transgenic wheat plants.

(a) Expression of *RDDK-Bar* determined by semi-quantitative RT-PCR in the wild-type (WT), and three independent transgenic lines that homozygous for the *RDDK-Bar* transgene (w2, w3 and w5). Levels of *TUBA-2A* transcripts were used as loading controls.

(b) Shld1-induced accumulation of RDDK-Bar fusion protein in the three independent transgenic lines. Immunoblotting with anti-HA antibody of total extracts from RDDK-Bar and WT plants treated with 10 μM Shld1 or mock solution for 8 hours. Ponceau S staining was used as a protein loading control.

(c) Shld1-induced bialaphos resistance of RDDK-Bar transgenic wheat as shown by CR assay. Leaf pieces of WT and RDDK-Bar transgenic wheat plants were excised and cultured separately in a 24-well plate with CR medium supplemented with (“+”) or without ( “-” ) 8 mg/L bialaphos and/or 10 μM Shld1. The plate was incubated in a growth chamber at 24°C with a light/dark cycle of 16 hours/8 hours. Photographs were taken three days after treatment.

**Table S1.** Transgenic lines generated in this study

| Plant species | Construct | Number of  T0 lines confirmed by PCR | T1 transgenic lines with 3:1 segregation of transgene | Homozygous T2 transgenic lines |
| --- | --- | --- | --- | --- |
| rice | RDDK-EGFP | 40 | e4, e5, e 6, e9, e10, e15, e16, e20, e35 | e4, e15^*^, e35 |
| rice | RDDK-Bar | 29 | b2, b5, b6, b7, b11, b13, b19, b22, b29 | b11, b13, b19^*^ |
| rice | RDDK-Pid3 | 27 | p2, p3, p4, p5, p6, p19, p23, p26 | p4, p5^*^, p6 |
| wheat | RDDK-GUS | 28 | g25, g29, g41, g44, g62 | g25^*^, g41, g44 |
| wheat | RDDK-Bar | 39 | w2, w3, w5, w9, w31, w85, w89, w100 | w2, w3, w5^*^ |

* The lines used for detailed analysis and data presented in the text.

**Table S2.** Sequences of the primers used in this work.

| Gene | Primer | Sequence |
| --- | --- | --- |
| *RDDK-EGFP* | Ub-Bgl II-for | AATTAAGCTTATGCAGATCTTCGTAAAGACCCTGAC |
|  | EGFP-EcoRI-rev | AATTGAATTCTTACTTGTACAGCTCGTCCATG |
| *RDDK-HA* | Ub-Bgl II-for | AATTAAGCTTATGCAGATCTTCGTAAAGACCCTGAC |
|  | MSBSK-HA-rev | ATATGGTACCAGGCCTTGTACAACTAGTACGCGTAGCGTAATCTGGAACGTCATATGGA |
| *Bar* | Bar-KpnI-for | AATTGGTACCATGAGCCCAGAACGACG |
|  | Bar-BamHI-rev | AATTGGATCCTCAGATCTCGGTGACGGGCAG |
| *GUS* | GUS-KpnI-for | ATATGGTACCATGTTACGTCCTGTAGAAAC |
|  | GUS-BamHI-rev | ATATGGATCCTCATTGTTTGCCTCCCTGCTG |
| *Pid3* | Pid3-SpeI-for | AATTACTAGTATGGCGGAGGGTGTTGTGGGCTCAC |
|  | Pid3-KpnI-rev | AATTGGTACCTTATTGAATCCTTTCTGCAGCCAACACAC |
| *RDDKHA-GUS* | Ub-HindIII-for | AATTAAGCTTATGCAGATCTTCGTAAAGACCCTGAC |
|  | GUS-EcoRI-rev | GCGAATTCTCATTGTTTGCCTCCCTGCTG |
| *RDDKHA-Bar* | Ub-HindIII-for | AATTAAGCTTATGCAGATCTTCGTAAAGACCCTGAC |
|  | Bar-EcoRI-rev | AATTGAATTCTCAGATCTCGGTGACGGGCAG |

**Cloning primers**

Note: The restriction enzyme sites are underlined.

| Gene | Primer | Sequence |
| --- | --- | --- |
| *RDDK* | DD-for | CCGGGATGCTTGAAGATGGAAAGAAAGTC |
|  | DD-rev | GGTTTTAGAAGCTCCACATCGAAGACGAG |
| *EGFP* | EGFP-for | CGGCGACGTAAACGGCCACAAGTTC |
|  | EGFP-rev | ACTTGTACAGCTCGTCCATGCCGAG |
| *Bar* | Bar-for | GCACCATCGTCAACCACTACATCGAG |
|  | Bar-rev | CCAGCTGCCAGAAACCCACGTC |
| *Pid3* | Pid3-for | ATGGCGGAGGGTGTTGTGGGCTC |
|  | Pid3-rev | TCCTCCCTCTTGAAGAGTACAGAGTCAG |
| *GUS* | GUS-for | GATTACCGACGAAAACGGCAAG |
|  | GUS-rev | AGGAACTGTTCGCCCTTCACTG |

**Primers for transgenic plant genotyping**

**Primers for qRT-PCR**

| Gene | Primer | Sequence |
| --- | --- | --- |
| *RDDK-EGFP* | Q-GFP-for | CCACATGCCACTCTCGTCTTCG |
|  | Q-GFP-rev | AGGGTCAGCTTGCCGTAGGT |
| *Os-UBQ5* | Q-OsUBQ5-F1 | GGCACCTTCATGGCCAACCAC |
|  | Q-OsUBQ5-R1 | CTACGCCTAAGCCTGCTGGTTG |

| Gene | Primer | Sequence |
| --- | --- | --- |
| *RDDK-EGFP* | rt-DD-for | GGAGTGCAGGTGGAAACCATC |
|  | rt-GFP-rev | GTTGCCGTCGTCCTTGAAGAAGATG |
| *RDDK-Bar* | rt-DD-for | GGAGTGCAGGTGGAAACCATC |
|  | rt-Bar-rev | TGCTTGTCTCGATGTAGTGGTTG |
| *RDDK-Pid3* | rt-DD-for | GGAGTGCAGGTGGAAACCATC |
|  | rt-Pid3-rev | CAAGAAGGCATGGATGCTCTC |
| *ACTIN1* | rt-ACTIN-for | CAGTCCAAGAGGGGTATCTTGAC |
|  | rt-ACTIN-rev | GGGCGATGTAGGAAAGCTTCTC |
| *TUBA-2A* | rt-TUB-for | ACCGCCAGCTCTTCCACCCT |
|  | rt-TUB-rev | TCACTGGGGCATAGGAGGAA |

**Primers for RT-PCR**
